# Editorial: Heart failure with preserved ejection fraction: Basic, translational, and clinical research

**DOI:** 10.3389/fphys.2022.1092009

**Published:** 2022-12-08

**Authors:** Fabien Brette, Pierre Dos Santos, Jean-Sebastien Hulot

**Affiliations:** ^1^ INSERM U1045, Université de Bordeaux, Bordeaux, France; ^2^ IHU LIRYC, CRCTB U1045, Pessac, France; ^3^ Phymedexp INSERM U1046, CNRS, Université de Montpellier, CHRU Montpellier, Montpellier, France; ^4^ Heart Failure Unit, Cardiology Department, Centre Hospitalier Universitaire (CHU) Haut-Lévèque, Bordeaux, France; ^5^ Université de Paris Cité, INSERM, PARCC, Paris, France; ^6^ CIC1418 and DMU CARTE, AP-HP: Assistance Publique—Hopitaux de Paris, PARCC, Hôpital Européen Georges-Pompidou, Paris, France

**Keywords:** heart failure, disease model, diastolic dysfunction, basic mechanisms, diagnosis, therapeutic target

Heart failure (HF) is a complex clinical syndrome that occurs as a consequence of structural or functional impairment of ventricular filling and/or ejection (Heidenreich et al., 2022). It is a worldwide condition with a high prevalence and mortality and is a frequent cause of recurrent hospitalizations. As per the most recent guidelines, HF is classified into three categories according to left ventricular (LV) ejection fraction (EF): HF with reduced ejection fraction (HFrEF) when EF is less than 40%, HF with preserved ejection fraction (HFpEF) when EF is greater than 50%, and the newly renamed HF with mildly reduced ejection fraction (HFmrEF) when EF is between 40 and 49%. Recent studies have reported a continuous increase in the proportion of patients with HFpEF, representing approximately 50% of all HF cases (Fayol et al., 2022). However, the mechanisms leading to HFpEF remain poorly understood.

Over the last 30 years, a plethora of data from animal models have improved our understanding of the pathophysiology of HFrEF at the molecular and cellular levels. Meanwhile, clinicians have developed and used different therapeutic strategies for treating HFrEF. Disappointingly, effective therapies for patients with HFrEF are not adequate, with the notable exception of sodium-glucose cotransporter two inhibitors (SGLT2i); their benefit for these patients was recently demonstrated (Anker et al., 2021; Solomon et al., 2022). Although the exact mechanism by which SGLT2i affect cardiac cells remains unclear and debatable (Dyck et al., 2022), recent evidence suggests a direct inhibition of sodium-hydrogen exchanger 1 (NHE-1). At the clinical level, impaired LV relaxation and filling is characterized by a decreased ventricular relaxation rate and an increased left ventricular chamber stiffness. More specifically, patients with HFpEF present an impaired diastolic reserve, characterized by an inadequate increase in myocardial relaxation and filling in response to situations requiring increased energy demand, such as exercise (Phan et al., 2009; Borlaug et al., 2011).

**FIGURE 1:**
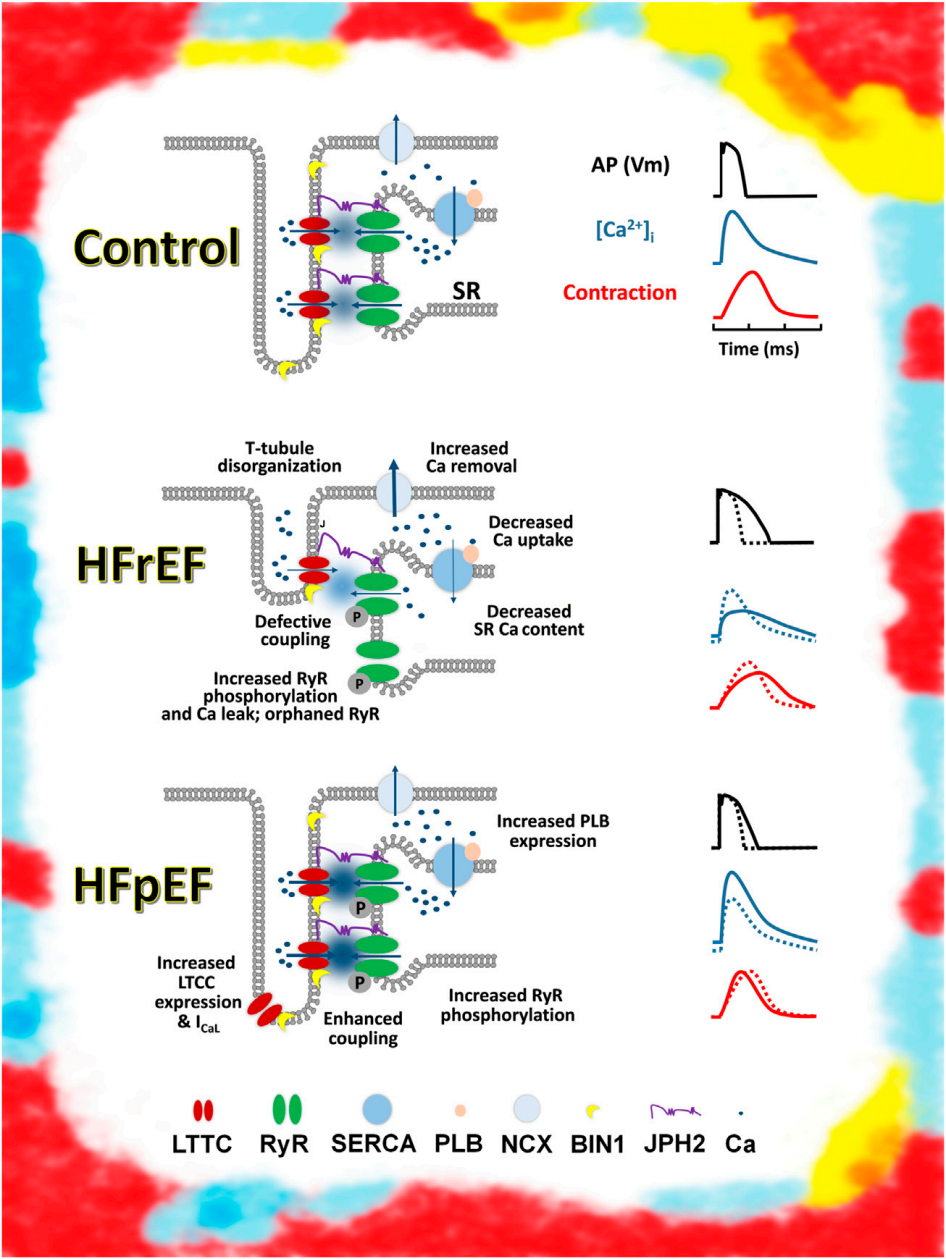
Schematic representation of excitation contraction coupling in control, HFrEF, and HFpEF cardiomyocytes Top: Control (healthy) and ECC (left). Schematic action potential, Ca transient, and contraction (right). Middle: major change observed in ECC in HFrEF cardiomyocytes (left). Change in action potential, Ca transient, and contraction, compared with a control (dashed lines) (right). Bottom: change observed in ECC in HFpEF cardiomyocytes by Kilfoil et al. (2020) (left). Change in action potential, Ca transient, and contraction, compared with a control (dashed lines) (right). Artistic rendering inspired by the work of Mark Titchner on billboards in the United Kingdom.

The mechanisms leading to impaired LV relaxation and filling remain obscure. They involve the consequences of multiple comorbidities and other contributing factors such as aging or lifestyle habits. Recent studies have shown that HFpEF is a heterogeneous syndrome, with dissimilar abnormalities observed at the cellular level in patients. For instance, recent studies have shown that disruption of the t-tubules in HFpEF is not involved, unlike in HFrEF (Frisk et al., 2021); the consequences also depend on the diabetes status as diabetic patients with HFpEF display impaired calcium handling, whereas patients with HFrEF do not (Hulot & Livrozet, 2021). This not only highlights the divergent pathophysiology between patients with HFrEF and HFpEF, but also within patients with HFpEF (Figure 1). More mechanistic studies are thus needed in animal, patient, or innovative models of cardiac relaxation (Kilfoil et al., 2020).

In this Research Topic, we present a series of articles from experts in various areas of HF.

The Research Topic starts with two articles on cardiorespiratory dysfunction and HFpEF.

In a review article, Toledo et al. discuss the possible effect of the activation of central chemoreflex pathways on autonomic function and its potential role in breathing disorders during HFpEF. By comparing to HFrEF, where most studies have been done (in patients and animal models), the authors highlight that enhanced central chemoreflex sensitivity, cardiac autonomic imbalance, and breathing disorders are all observed in experimental animal models based on non-ischemic HFpEF. However, the precise mechanism(s) responsible for central chemoreflex sensitization, autonomic imbalance, and respiratory disorders in HFpEF are not yet clearly established.

As Toledo et al. ask for further studies on cardiorespiratory dysfunction, Jones et al. investigate the relation between obstructive sleep apnea and obesity in animal models. They show that caloric restriction can prevent obesity-induced LV hypertrophy. This protects against intermittent hypoxia induced remodeling, independent of leptin signaling. Clinical relevance of these observations needs additional investigations.

A second review article focuses on endothelial dysfunction as a critical player of HFpEF development. The review by Cornuault et al. aims to present and discuss evidence supporting a causal role of endothelial dysfunction in the pathophysiology of HFpEF in patients and animal models. Endothelial cells dysfunction can alter cardiomyocytes functions such as survival, contractility and metabolism. The authors conclude that at molecular and cellular levels, endothelial to cardiomyocyte crosstalk is still poorly understood and new animal models of HFpEF should bring some clues regarding pathophysiology.

In this Research Topic, an article by Lamonzie et al. provides a new aspect on imaging diagnosis. Cardiac magnetic resonance imaging (MRI) is the gold standard for evaluating myocardial function, volumes, and scarring. Currently, gadolinium-based contrast agents are widely used to improve the sensitivity and specificity of imaging. As an extracellular agent, gadolinium is a powerful tool for imaging scarring and highlighting intravascular and extracellular spaces. Manganese-enhanced MRI (MEMRI) has emerged as a complementary approach, enabling intracellular myocardial contrast imaging that can identify functional myocardium through its ability to act as a calcium analog. An important issue is the possible cardiac toxicity of manganese. Lamonzie et al. provide a comprehensive evaluation of its toxicity. At the whole heart level, micromolar concentrations of manganese, up to 25 μM, have no impact on cardiac function. At the cellular level, a small decrease in the calcium transient amplitude and action potential duration are observed, mainly due to a block of L-type Ca^2+^ channels. MnCl_2_ at a concentration of 25 µM decreases T1 in MRI experiments, demonstrating that this concentration is sufficient for using manganese as a contrast agent. The authors conclude that as MEMRI has the potential to monitor changes in intracellular Ca^2+^ homeostasis at the whole heart level, it should optimize patient diagnosis and therapeutic decision-making by providing visualization of potentially arrhythmic locations.

The last research article uses numerical modelling to investigate the role of the Purkinje fiber-ventricle junction on pro-arrhythmic events. Jian et al. demonstrate that HF-induced ionic remodeling reduced conduction safety and increased tissue vulnerability to the genesis of unidirectional conduction blocks. The Purkinje fiber-ventricle junction can form a potential substrate for the genesis of conduction failure that leads to re-entry. The study by Jian et al. highlights the utility of single cell and multiscale modeling of cardiac electrophysiology as an important tool to widen the understanding of underlying mechanisms at cellular and tissue levels.

In conclusion, this Research Topic covers a broad range of HFpEF at different levels. Cleary, more basic, translational, and clinical research is needed to delineate the complexity of the mechanisms involved in HFpEF and their interactions. Given the multidimensionality of the HFpEF syndrome, new experimental models, which represent clinical situations, are required. In terms of patients, in depth imaging as well as genomic, proteomic, and metabolomic analyses will help for a better characterization. Both human and animal models will contribute to identifying potential therapeutic targets. We hope that this Research Topic encourages further investigation of these exciting areas.
